# Neurasthenia from the Standpoint of a General Practitioner

**Published:** 1899-09

**Authors:** C. W. Coulter

**Affiliations:** Oil City, Pa.


### NEURASTHENIA FROM THE STANDPOINT OF A GENERAL PRACTITIONER.

By C. W. COULTER, M. D., Oil City, Pa.

IN THE class of neuroses, with its various manifestations, there is none so perplexing at times to the general practitioner, as the one upon which I shall speak briefly on this occasion. To listen to neurastheniacs enumerating their ailments is to say the least wearisome, yet there is no complaint with which one must exercise more patience or give a more attentive ear than this, for each symptom enumerated by a patient thus afflicted only serves to confirm the true diagnosis of the disease; for surely it is a disease, a perversion from the normal, a poorly balanced condition of the great entity called physiological life.

This great something which we call physiological life, and which has been defined as force, or a constant and continuous adjustment of internal relations to external relation, lies in the nerve cell. If this accommodation of adjustment be disturbed, derangements are sure to follow, which will be in proportion to the extent of this failure of accommodation in adjusting the equilibrium of nutrition. According to Cajal, the psychic nerve cell, the trophic nerve cell and the motor and sensory nerve cell, are united by contact, and since nervous conduction is by contiguity of nerve cells and may be over a long distance of contiguous nerve cells, one can partially comprehend why so many and varied disturbances may arise from a failure of accommodation of adjustment, or a failure of nutrition of the nerve cell. When disturbances of this character exist we have a condition which is known by the distinctive name of neurasthenia.

Neurasthenia is perhaps more common in the United States than in any other country; in fact, it has been called the American disease. The American people are prone to neurotic troubles, their fast living, long hours of work, from which they either refuse or are unable to divorce themselves, often retiring late at night, with their minds occupied with cares that should infest only the day. The expenditure of nerve energy is almost constantly greater than the production and as a consequence, a nervous breakdown occurs, and tired nature demands a rest. Heredity plays a very important part in the etiology of neurasthenia. Those who inherit a nervous system weak in energy are extremely susceptible to neurasthenia, while those who inherit the opposite or a nervous system full of force are almost invulnerable to it. Many are the conditions which are considered etiological of neurasthenia, among which I may mention overwork, mental emotions—as worry, fright, sorrow—masturbation, sexual excess, and occupation of various kinds, but chiefly those of an indoor nature. Persons of an active brain, if nervously predisposed, or of a nervous temperament, or deficient in nerve energy, are especially prone to neurasthenia. Let their occupation be what it may, their power of adjusting the expenditure and production is not even; equilibrium with them in this respect seems to be an impossibility. Perhaps if their environment could be somewhat changed to assist in the conserving of what little nervous energy they are capable of producing and the expenditure of the same lessened, results with them would be different.

To classify the symptomatology of this disease is next to an impossibility, but it may be grouped in a general way as relating to the digestive, vascular, motor, sexual, mental and sensory systems. The digestive symptoms are not of a regular type in all cases. There may be present in a given case one or more of the following symptoms—namely, gastric indigestion, intestinal indigestion, variable appetite, coated tongue, constipation, or an irregular condition of the bowels. Under this class of symptoms, I especially wish to invite attention to those pertaining to the abdomen, which are abdominal tenderness, colicky pains and the discharge of considerable mucus. The abdominal symptoms thus indicated are strongly symptomatic of neurasthenia.

The vascular symptoms are manifested by irregular actions of the heart, palpitation, tachycardia, and there may be bradycardia; hot flashes, coldness of the extremities and a mottled condition of the skin, due to vaso-motor disturbances, and polyuria may be present. The motor symptoms are mostly static in character and are general. The sexual are impotence, nocturnal emissions, spermatorrhea and prostration after coitus. The mentality of the neurastheniac is one of static depression. These patients lack power of mental concentration, are irritable, have a dread or fear of present or future happenings, which is undefinable; in fact, life to the neurastheniac is dotted with mythical absurdities and they are, in a word, pessimistic.

The sensory symptomatology is an important one; pains are complained of which are at times obscure and ill-defined, but the pain that is generally present in these cases and to which I attach considerable importance, is located at the base of the brain with a tendency to radiate down the spine. Upon examination there will be found one or more points of spinal tenderness. Vertigo occasionally presents itself and local anesthesia or hyperesthesia may be present. Sleep is generally extremely irregular, insomnia being a frequent and obstinate condition to deal with.

Nearly all diseases classed as neuroses are so-called function disturbances and are without any known pathology; in fact, many practitioners adhere to the belief that functional derangements have no special pathological significance, but at this late day we cannot successfully claim that disorder of function has no anatomical correlate.

The physician of superficial examination and study is very liable to look upon these cases as hysterical or hypochondrical, usually administering some measures directed to meet his views, paying no further heed to them. The quack and charlatan are very apt to reap the benefit of such errors. That neurasthenia, hysteria and hypochondriasis are separate and distinct conditions there can be no question, admitting, however, that the neurastheniac may at times become hysterical. The diagnosis must be differentiated from lithemia, insipient melancholy, hysteria, hypochondriasis and the stigmata of degeneration.

It is not the purpose of this paper to go into the details of a differential diagnosis of these various manifestations of abnormal conditions, but I will dwell for a moment comparatively upon neurasthenia and lithemia. Believing, as I do, that neurasthenia is an inability to preserve the equilibrium of adjustment between production and expenditure of nerve energy necessary to the proper nutrition of the nerve cell, and that lithemia is a faulty metabolism due to insufficient oxidation of the ingesta, I can readily understand that the neurastheniac may become lithemic, and that a lithemic may suffer from neurasthenia; but if the careful observer will follow the two conditions clinically, he will soon discover that they are separate and distinct conditions. I am fully aware that some observers in the study of clinical medicine hold that many, if not all, cases of neurasthenia originate in autointoxication, claiming that autointoxication is produced by over-fatigue, sexual excesses, mental anxiety and the like, and that neurasthenia is more common among the indolent than energetic persons. Such theory does not accord with my clinical observations. A case from my clientèle will better illustrate than any theory I might advance.

Miss M., aged about 34, came under my observation and care a few years ago. Her cares were many, as she was compelled to earn her own livelihood. She was superintendent of a hospital, had a neurotic temperament, but always enjoyed excellent health up to the time of which I write. In her capacity as superintendent of a hospital she was nurse, head nurse, matron, housekeeper, bookkeeper, cashier and general overseer of the institution, in addition to superintending the training school for nurses. After a long siege of a typhoid fever epidemic, in which she had many extra cares, which were very arduous, she collapsed and soon consulted me for insomnia. Still later I was called to see her again, and upon careful examination found her suffering from the following symptoms: low spirits, seeking seclusion, skin mottled from vascular disturbances, hands and feet cold, headache, with a dull throbbing pain at the occiput, occasionally radiating down the spine, a few points of spinal tenderness, insomnia, loss of interest in her work, loss of appetite with disgust for food, tongue slightly coated—all the symptoms of atonic indigestion—slight tenderness over the abdomen, but more especially over the cecal region, considerable abdominal pain, bowels slightly constipated, fecal discharges at times quite green, containing considerable mucus. Her pulse was small and weak, the heart examination revealed that organ healthy but weak as to force and normal as to frequency; there was no arythmia, no tachycardia, in fact no departure from the normal healthy heart other than that of being weak as to force. Temperature at no time during her disability was found other than normal. After this continued illness, extending over a period of about four years, at times better and others worse, she has now regained the power of adjustment between production and expenditure of nerve energy and is well.

Careful observation of this case failed to demonstrate lithemia or autointoxication, but all the symptoms pointed to nerve exhaustion. There is, it is true, a similarity in the symptomatology of the two diseases, which, I confess, is at times confusing but with our modern methods of diagnosis such as urinalysis and the like, the diagnosis becomes comparatively easy. Habit, temperament, family history, environment assist materially in making a diagnosis. All the cases of neurasthenia coming under my observation have occurred in persons who were active and energetic, while in lithemia the converse is true, hence we assert persons of indolent habits and phlegmatic temperament are seldom if ever subject to neurasthenia, while they are peculiarly liable to lithemia; on the contrary, energetic persons, of active habits and nervous temperament are peculiarly susceptible to neurasthenia, but rarely so to lithemia.

A patient under my care at the present time, presented at first consultation the following symptoms:

Mrs. D., aged about 40. Married. Mother of three small children. Previous to marriage she had been a school teacher and had always enjoyed a fair degree of health until about one year ago, when she broke down. She first consulted me on account of a severe cough, which was alarming her family considerably; the cough was almost incessant; there was no expectoration and no fever; examination of the lungs revealed nothing of an abnormal character. There was tachycardia, the heart running at 130 and over. She is naturally spare but seemed to be somewhat emaciated. There was considerable vaso-motor disturbance, as evidenced by the lowered condition of the cutaneous or peripheral circulation. Her history shows her to be of a neurotic family. She is very energetic and of a sunny disposition. Her household duties were too much for her; expenditure of nerve energy in her case exceeded production of the same and she was compelled to seek relief. Under treatment this patient improved rapidly; the cough, which was a neurosis, entirely disappeared, the circulation improved, the tachycardia ceased, the pulsations dropping to 70. About nine months after this she again called upon me for "numb spells and dizziness," which she says frighten her very much. Upon examination I found the tachycardia had returned, also the vasomotor disturbance, but not the cough. Under treatment this patient is again improving.

These two cases cited illustrate the variableness of the symptoms in the same disease. That a systematic train of symptoms can be arranged as a means of diagnosis in neurasthenia is scarcely possible. Each case is a study in and of itself. One will present symptoms of atonic indigestion, or what we might term digestive neurasthenia; another insomnia, with its attendant mental disturbances; another with sexual neurasthenia, and another with abdominal symptoms and the like. These groups may be predominating symptoms in as many different cases, or we may find them all present in a single case.

An eminent gynecologist once said it would be well for the surgeon to remember that women had other organs besides a uterus, ovaries and tubes. To this I would add that it would be wise to look well to the nervous system ere mutilating or unsexing the unfortunate sufferer. A case came to my knowledge a few years ago of a woman who had been ill of an obscure trouble for some time. Her family relations were not such as conduced to her happiness and contentment of mind. After failing of cure by her family physician, she visited one and another until a so-called gynecic surgeon informed her her trouble was all caused from the ovaries, and advised they should be removed, promising a cure; she submitted, but two years afterward she was still searching for some doctor who could cure her of neurasthenia.

Again, a young woman of my acquaintance became ill and was treated at first by a general practitioner of considerable ability. After a time she became convinced he could not help her, and sought relief from a physician in a neighboring town. This physician discovered she had a retroverted uterus, which he believed to be the cause of all her trouble. About this time she was disappointed of marriage and became worse. Finally, a homeopathic physician was consulted, but he, like the others, failed to cure and then all doctors were abandoned. For over a year afterward she was practically helpless, when the christian scientists convinced her of their ability to cure her, and succeeded in producing psychic influence enough to relieve her neurasthenia. Such cases as these point a moral and should be studied carefully.

Therapeutic indications in this disease are limited to the requirements of the individual case. The line of therapy must be decided upon from the history and present condition of the patient. We may classify our treatment as follows: psychic, environmental, climatic and medicinal. In the psychic treatment the physician must have the absolute and perfect confidence of his patient, else his power will be *nil.* Hard, indeed, will be the task of the physician who attempts to treat a case of neurasthenia without first securing the patient's confidence. The physician must be able to direct the mind of the sufferer; he must be patient, kind, firm and enduring; he must satisfy the patient's mind of his ability to treat the case in a masterful way and then back it up with the skill that is convincing.

These patients should be free from care and worry; mental occupation, however, of some kind is necessary; if not their thoughts will continually revert to themselves, and the belief will grow upon them that their malady is incurable, hence such a morbid condition must be continually guarded against by the physician.

To apply environmental therapeutics is, sometimes, one of the most difficult of tasks, for not only the patient but the patient's friends have to be considered. Very frequently financial conditions will not afford a change of environment, and when such is the case, the physician starts with a handicap. The influence of sympathising friends, unpleasant home life, arduous duties and many other conditions of environment, which the alert practitioner will recognise, are conducive to a certain chronicity of the disease, to correct which is necessary and which requires tact and diplomacy on the part of the physician.

The type of the disease and the individuality of the patient must govern the physician in advising rest. If myasthenia, anemia and loss of flesh be present, rest in bed for a period of three or four weeks with forced feeding is advisable. Massage and exercise are both indicated in a number of cases for their metabolic advantages.

The diet of the neurasthenic should be very liberal, only such articles of food being forbidden as will be difficult of digestion. No routine plan can be adopted in the treatment of these cases. Atonic indigestion is almost a constant symptom and if improper food be given, fermentation with its consequent autointoxication is almost sure to follow. Fresh garden vegetables, as peas, beans, celery, spinach and the like, are wholesome and can generally be advised with advantage.

In selecting a climate for neurastheniacs, I have found the salubrious air of the sea shore more invigorating than the pure ozonised air of the mountains. If the patient cannot afford a rest at the sea shore, the next best plan is a change from the climate of home to some place as distant as possible, where a psycho-climato-environmental change can be thorough.

In the medicinal treatment of this disorder I have very little confidence, and yet I do not wish to be understood as saying that there is nothing in drugs in the treatment of the disease. I do not believe, however, that drugs alone will restore the equilibrium of the nervous system when it is once disturbed. The symptomatic treatment is rational and reasonable. Emaciation, anemia, insomnia, cardiac disturbances, head pressure, indigestions, autointoxications and the like can all be materially improved by judicious medication. I have purposely omitted many points in the therapy of neurasthenia, my object being simply to call attention to the main points in the disease.

In conclusion permit me to reiterate, each case of neurasthenia must be diagnosticated individually and not from any single group of symptoms, and the therapeutics must be arranged to meet the demands of individual cases. In other words, each and every case of neurasthenia is a study in and of itself.

